# Dual‐Isolation and Profiling of Circulating Tumor Cells and Cancer Exosomes from Blood Samples with Melanoma Using Immunoaffinity‐Based Microfluidic Interfaces

**DOI:** 10.1002/advs.202001581

**Published:** 2020-08-19

**Authors:** Yoon‐Tae Kang, Thomas Hadlock, Ting‐Wen Lo, Emma Purcell, Anusha Mutukuri, Shamileh Fouladdel, Monica De Silva Raguera, Heather Fairbairn, Vasudha Murlidhar, Alison Durham, Scott A. McLean, Sunitha Nagrath

**Affiliations:** ^1^ Department of Chemical Engineering and Biointerface Institute University of Michigan 2800 Plymouth Road, NCRC B10‐A184 Ann Arbor MI 48109 USA; ^2^ University of Michigan‐Michigan Medicine 1910 Taubman Center 1500 E. Medical Center Drive Ann Arbor MI 48109 USA; ^3^ Roger Cancer Center University of Michigan 1500 E Medical Center Ann Arbor 48109 USA; ^4^ Michigan Medicine Otolaryngology Clinic 1910 Taubman Center 1500 E. Medical Center Drive Ann Arbor MI 48109 USA

**Keywords:** circulating tumor cells, dual isolation and profiling, melanoma, microfluidics, tumor exosomes

## Abstract

Melanoma is among the most aggressive cancers, and its rate of incidence continues to grow. Early detection of melanoma has been hampered due to the lack of promising markers for testing. Recent advances in liquid biopsy have proposed noninvasive alternatives for cancer diagnosis and monitoring. Circulating tumor cells (CTCs) and cancer‐exosomes are gaining influence as promising biomarkers because of their cancer‐associated molecular markers and signatures. However, technologies that offer the dual‐isolation of CTCs and exosomes using a single sample have not been thoroughly developed. The dual‐utilization OncoBean (DUO) device is conjugated with melanoma specific antibodies, MCAM and MCSP, enabling simultaneous CTC and exosome isolations. Using blood samples from patients, CTCs and exosomes are specifically isolated from a single sample and then undergo molecular profiling for comprehensive study. Melanoma patients have 0–17CTCs mL^−1^ and 299 µg exosomal protein mL^−1^ while healthy donors display fewer than 2CTCs and 75.6 µg of exosomes mL^−1^, respectively. It is also demonstrated that both markers express melanoma‐associated genes using multiplex qRT‐PCR to test for expression pattern of a 96 gene panel. The dual isolation and molecular characterization will allow for further research into melanoma to identify viable markers for disease progression and treatment efficacy.

## Graphical abstract

Circulating tumor cells (CTCs) and cancer exosomes are gaining influence as promising biomarkers in liquid biopsy, however, technologies that offer the dual‐isolation of CTCs and exosomes have not been developed. Here, a microfluidic device is devised conjugated with melanoma cell adhesion molecule (MCAM) and melanoma‐associated chondroitin sulfate proteoglycan (MCSP) for isolation and molecular profiling of both melanoma CTCs and exosomes.

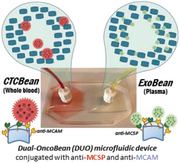

## 1 Introduction

Melanoma is one of the most aggressive cancers whose incidence rate keeps increasing with over 300 000 new cases ^[^ ^1^ ^]^ reported globally and 100 350 cases projected in the United States in 2020.^[^ ^2^ ^]^ Due to the lack of promising markers to predict the disease and onset of metastasis, little progress has been made toward the early detection and evaluation of treatment efficacy. Early detection of melanoma is critical as early stage localized melanoma patients have a 98% survival rate^[^ ^3^ ^]^ while patients who develop metastatic melanoma can expect a 5–19% 5‐year survival rate.^[^ ^4^ ^]^ Despite significant research into melanoma metastasis, there is still a distinct lack of predictive biomarker, which has in turn led to minimal progress towards reducing the mortality rate.^[^ ^2^ ^]^

Recent advances in the field of liquid biopsies have proposed alternatives for diagnosing disease with the merits of enabling continuous monitoring and non‐invasiveness. Circulating tumor cells (CTCs) and cancer‐derived exosomes have recently evolved as promising biomarkers for many cancer types, including lung, prostate, and breast cancers, where the cancer‐associated molecules correlate to cancer progression, overall survival, and treatment efficacy. CTCs, shed from the primary tumor site into blood vessels, are a known medium of secondary tumor occurrence or metastasis.^[^ ^5^, ^6^ ^]^ Recent studies have shown that the presence and number in peripheral blood is associated with metastatic relapse and tumor burden, as well as the aggressiveness of cancer and susceptibility to applied anticancer drugs.^[^ ^7^, ^8^ ^]^ CTCs in melanoma have also demonstrated the ability to reflect tumor susceptibility to immune checkpoint inhibitor treatment.^[^ ^9^ ^]^

Despite their advantages, CTC‐based melanoma diagnostic or prognostic tests have not been applied to clinical practice. This is largely because the only FDA cleared device, CellSearch, is largely ineffective for isolation melanoma CTCs. CellSearch, and similar CTC isolation devices, have largely been developed to isolate CTCs using an antibody against epithelial cell adhesion molecule (EpCAM), which is downregulated on melanoma CTCs.^[^ ^10^ ^]^ Since 2011, the CellSearch system has improved for melanoma CTC detection through the development of the MelCTC kit which replaces anti‐EpCAM with a more melanoma specific melanoma cell adhesion molecule (MCAM) followed by detection using an melanoma‐associated chondroitin sulfate proteoglycan (MCSP)/chondroitin‐surface proteoglycan 4 (CSGP4)/human high molecular weight‐melanoma‐associated antigen (HMW‐MAA) immunostaining panel.^[^ ^11^, ^12^ ^]^ This research use only kit has yielded improved results over the original anti‐EpCAM based CellSearch system but 60% of enrolled patients had no CTCs detected.^[^ ^13^, ^14^ ^]^ Similar to the MelCTC kit, melanoma CTC isolation using immunomagnetic beads conjugated with two melanoma specific antibodies, anti‐MCSP and anti‐MCAM, has proven to be effective in isolating over 80% of CTCs in stage IV melanoma patient blood samples, however its median CTC count was less than 2 CTCs per ml blood (1.78 CTCs mL^−1^).^[^ ^9^ ^]^ CTC concentration in melanoma patients using previous methods is often very low between 0 and 36 per 7.5 mL blood,^[^ ^13^ ^]^ and thus insufficient to perform diverse clinical studies such as drug screening or functional in vitro*/*in vivo studies.^[^ ^15^ ^]^ In order to overcome this drawback, simultaneous isolation of more than one circulating marker from same patients will allow for further understanding of melanoma reflecting each circulating marker's characteristic.

Along with CTCs, exosomes, nanoscale extracellular vesicles (EVs) actively secreted from malignant cells for cell‐to‐cell communication, have been used for cancer studies and diagnosis. Compared to CTCs, these vesicles are known to be more stable and abundant in body fluids, which facilitates cancer studies.^[^ ^16^, ^17^, ^18^ ^]^ Recent studies of cancer‐derived exosomes have discovered some of the important roles they play, especially in tumor progression, such as the transformation of neighboring cells, acquisition of drug resistance, and transfer of tumor‐associated information to other cells.^[^ ^19^, ^20^ ^]^ However, a lack of technologies to isolate and characterize tumor‐specific exosomes has minimalized their use in clinical settings. Thus far, standard exosome isolation methods, such as ultracentrifugation,^[^ ^21^, ^22^ ^]^ polymer‐based exosome isolation kits,^[^ ^23^ ^]^ and immunoaffinity based isolation using antibodies against exosomal surface proteins,^[^ ^24^, ^25^ ^]^ have been utilized for tumor exosome isolation. However, novel specific isolation methods to enrich for specifically tumor‐derived exosomes are urgently needed.^[^ ^26^, ^27^ ^]^ In addition to the research efforts toward melanoma CTCs, progress toward the isolation of melanoma‐specific exosomes has largely been made by introducing alternative antibody‐capture methods. Sharma et al. recently provided a way to extract melanoma specific exosomes from plasma samples using a combination of isolation methods including size exclusion chromatography and magnetic beads conjugated with antichondroitin surface proteoglycan 4 (CSPG4), which targets melanoma cells and exosomes.^[^ ^28^ ^]^ They verified the specificity of isolated exosomes using FACS and showed that the subset of EVs they captured were melanoma specific and related to immune suppression. Capture strategies incorporating large antibody cocktails including MCSP and MCAM have consistently shown higher results than single antibody isolation platforms.^[^ ^29^, ^30^ ^]^ However, these previous systems still need multiple isolation and incubation procedures, which hinder an easy isolation of circulating markers. Also, low capture efficiency and low sensitivity in the isolation of melanoma circulating markers has demonstrated a need for improvements, such as antibody cocktail optimization and its incorporation into microfluidics systems, which facilitate highly sensitive isolation from a limited volume of sample.^[^ ^31^, ^32^, ^33^ ^]^

Here, we devised the dual utilization of OncoBean (DUO) microfluidic device conjugated with melanoma‐specific antibodies, MCAM and MCSP, for capturing circulating markers in an immunoaffinity manner and applied this device to the isolation of both melanoma CTCs (MCTCs) and melanoma exosomes (MExos). As both markers originate from the same tumor sites, coexpression of surface markers allows for identical enrichment strategies.^[^ ^34^, ^35^ ^]^ Dual marker isolation using the DUO can yield improved insights due to the distinctive roles they might play in melanoma progression. Thus, coisolation and analysis of both markers from a single sample could aid in our understanding of the complexities in melanoma progression/diagnosis and be useful for better diagnosis and monitoring of individual patients’ clinical status. To this end, MCTCs and MExos are specifically isolated from patient whole blood samples by identical devices before undergoing molecular profiling. The radial flow‐based microfluidic device provides all the benefits of traditional immunoaffinity‐based microfluidic devices while allowing for high sample throughput.^[^ ^36^, ^37^, ^38^ ^]^ The inclusion of both isolation modules in a single platform provides significant convenience and allows room for future optimization such as attachment of our labs previously reported inertial force differentiating sample separation device for rapid and efficient multimarker analysis.^[^ ^39^ ^]^ To the best of our knowledge, dual isolation of MCTCs and MExos from single samples using a single platform has not been studied yet. This novel device and dual‐profiling of melanoma markers will enable a more comprehensive understanding of the disease, allowing for enhanced clinical decisions in the future.

## 2 Results and Discussion

### 2.1 Strategy for Dual Isolation of MCTCs and MExos Using DUO Microfluidic Device

**Figure 1:**
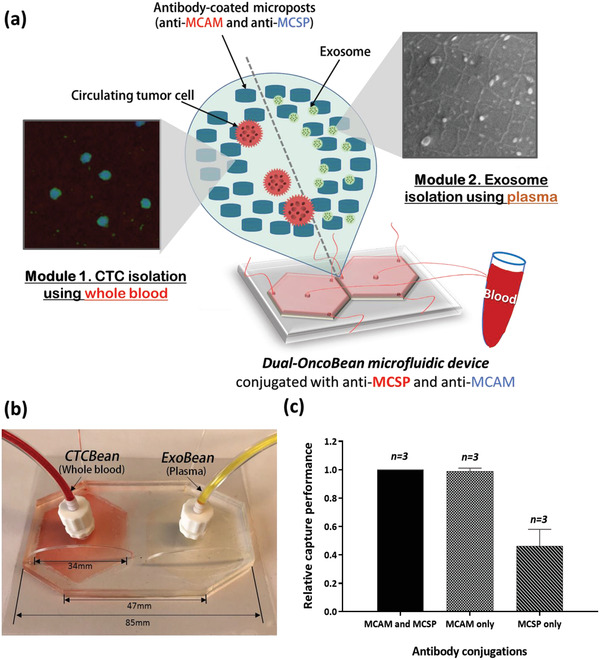
Dual‐isolation of circulating tumor cells (CTCs) and cancer exosomes via dual‐utilization of OncoBean (DUO) microfluidic device: a) schematic diagram of dual‐isolation of CTCs and exosomes from melanoma patient blood samples; b) fabricated DUO microfluidic device with dimensions; c) relative isolation performance of melanoma cancer cells with two different melanoma antibodies and their combination.

The advantages of the microfluidic OncoBean device have been described in our previous studies mostly involving CTC isolation.^[^ ^37^, ^38^ ^]^ Briefly, the OncoBean is a radial flow‐based microfluidic device with bean‐shape microposts functionalized with antibodies to capture targets. The radially placed micropost design specifically captures target cells expressing different antigen levels even using high flow rates of up to 10 mL h^−1^. For this work, two OncoBean modules are combined: one to isolate MCTCs and one MEXOs that were optimized for each marker type (**Figure** **1**b). To isolate MCTCs, first module, CTCBean, was originally conjugated with one of two different melanoma specific antibodies, anti‐MCAM and anti‐MCSP, or a combination of the two. Antibodies against MCSP and MCAM were chosen as they are the dominant cell surface proteins on MCTCs.^[^ ^9^ ^]^ In order to decrease nonspecific bindings, all devices were blocked with bovine serum albumin (BSA) solution after antibody conjugation. We compared capture performance between three different antibody combinations using an MCTC model sample (SK‐MEL‐103). We found that MCAM captures more melanoma cells than MCSP. The MCAM and MCSP combination showed similar capturing performance as MCAM on its own (Figure 1c). However, the use of both antibodies was chosen in order to isolate various melanoma subtypes including MCSP overexpressed melanoma cells; MCSP expression has been linked with tumor invasion and serves as an indicator of poor prognosis in patients.^[^ ^9^, ^29^, ^40^ ^]^ The specificity of MCSP and MCAM antibodies also allows for the targeting of specifically melanoma cancer‐derived exosomes for isolation, as over 85% of melanomas highly express MCSP.^[^ ^30^ ^]^ The second module, ExoBean, with MCAM/MCSP antibody conjugation, was also examined using melanoma patient plasma samples following passage through 200 nm filter (S1, Supporting Information). From this study, we found that ExoBeans conjugated with the MCAM/MCSP cocktail are capable of isolating MExo‐like vesicles more efficiently and more specifically compared to our device conjugated with the most common exosome antibody, anti‐CD63 (Figure S1, Supporting Information). Following both capture antibody optimization studies, the anti‐MCSP/MCAM cocktail was chosen as the optimized conjugation for isolation of both circulating markers and used in all future studies. After the verification of MExo isolation, we optimized the flow rate for exosome isolation (Figure S2, Supporting Information) with 1 mL h^−1^ showing the best recovery rate of exosome based on a protein quantity. This flow rate was then used in our clinical studies.

### 2.2 Dual Isolation of MCTCs and MExos Using Model Samples

**Figure 2:**
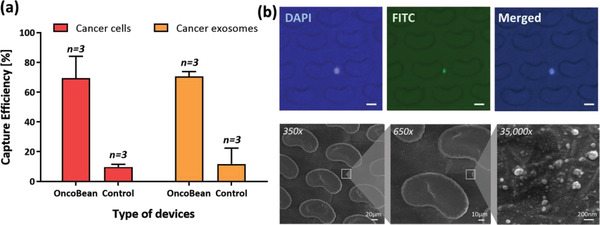
Isolation of circulating melanoma markers using the MCAM/MCSP functionalized DUO device: a) capture efficiencies of melanoma cells and exosomes from melanoma cell lines, SK‐MEL‐103, compared to control DUO device without antibody conjugation; b) isolated spiked cancer cells stained by DAPI and CellTracker green (top) and cancer extracellular vesicles on the device (bottom).

Capture efficiencies of the DUO device for both MCTCs and MExos were tested using SK‐MEL‐103 cells spiked in whole blood and SK‐MEL‐103 derived exosomes spiked in PBS buffer, respectively. The melanoma cell line was prefluoresced with CellTracker Green to facilitate counting the cells before and after isolation. For exosome experiments, we spiked a known number of purified exosomes into filtered PBS and measured the concentration before and after ExoBean isolation to evaluate capture efficiency. As shown in **Figure** **2**a, the DUO device demonstrated high capture efficiencies for both model sample CTCs and exosomes. The DUO device captured 70% of spiked cancer cells, as well as 75% of exosomes. This capture efficiency exceeds that of previously reported systems, such as that from Aya‐Bonilla et al. whose two‐stage platform produced a 55% capture efficiency in spiked melanoma CTC samples.^[^ ^41^ ^]^ Our DUO system also displayed this selective capture ability even when processing densely populated samples of 1000 cells mL^−1^, while the commercially available MelCTC CellSearch kit delivered a similar 74% capture efficiency but maxed out at only 160 cancer cells mL^−1^.^[^ ^14^ ^]^ The significant quantity of CTCs and exosomes captured by the DUO device allows for thorough analysis of disease progression through enumeration and RNA profiling. Nonfunctionalized control devices captured significantly reduced numbers of spiked MCTCS (8%) and MExos (15%). These nonzero efficiencies are likely due to nonspecific bonding within the chamber, as well as unintended sized based capture by the microposts within the device.

### 2.3 Isolation and Evaluation of Circulating Markers from Clinical Samples

**Figure 3:**
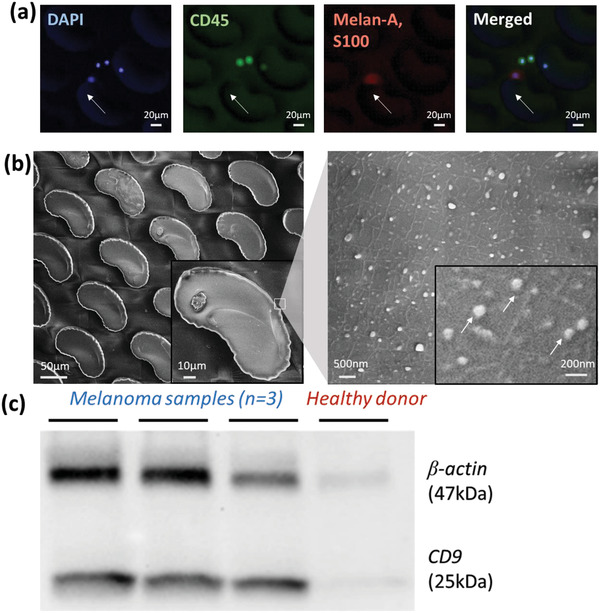
Evaluation of isolated melanoma circulating tumor cells and melanoma‐associated exosomes by MCAM/MCSP functionalized OncoBean devices; a) circulating melanoma cell stained by DAPI(nucleus), CD45(leukocyte), and cocktail of Melan‐A and S100 (melanoma); b) scanning electron microscopy image of isolated exosome‐like vesicles on the OncoBean device; c) western blot analysis of exosomes isolated by DUO (ExoBean).

For clinical evaluation of our ExoBean system, we enrolled 15 stage I–IV melanoma patients for quantification and characterization of blood borne MCTC and MExo markers. Both MCTCs and MExos were isolated from patient peripheral blood samples using the DUO device with melanoma‐specific capture antibodies and streamlined processing procedures. Whole blood and prefiltered plasma was used for MCTC and MExo isolation, respectively. MCTCs were isolated and enumerated using immunofluorescence for four staining markers: DAPI, CD45, and a S100 and melanoma antigen, Melan A. Positive identification of nucleus and melanoma cells was confirmed by DAPI and S100‐MelanA, respectively, with CD45 distinguishing white blood cells. Each CTCBean microfluidic device was imaged using fluorescence microscopy and enumerated were DAPI+/S100+, Melan A+/CD45‐ were counted as MCTCs. **Figure** **3**a shows a representative image of an MCTC captured on a single bean post along with several leukocytes distinguished by a lack of Melan A/S100 fluorescence. Isolation of MExos was carried out from the same set of patient samples as MCTCs. Due to the small size of exosomes, we used a scanning electron microscope (SEM) to visualize the MExos captured by an ExoBean. The SEM results verified the isolation of vesicles on the device surface and that the size of vesicles are in the range of extracellular vesicles (Figure 3b). In order to confirm that the vesicles isolated on the ExoBeans are exosomes, we used western blot analysis to verify the expression of exosomal markers. This western blot analysis was performed using three melanoma plasma samples and one healthy donor sample. As shown in Figure 3c, we verified positive bands for the exosomal marker CD9 and general housekeeping protein ß‐actin in the three melanoma patient samples tested, but not in the healthy donor. As the MCAM/MCSP isolation specifically targets melanoma specific circulating markers, the increased presence of both general exosomal proteins (CD9) and general housekeeping proteins (ß‐actin) found in melanoma patient samples would indicate that we are specifically capturing a subset of exosomes that is displaying the target marker set. This result indicates the presence of exosomes following MCAM/MCSP isolation of melanoma patient samples but not healthy donors, and shows that our ExoBean device specifically targeted and isolated MExos from plasma. For comparison studies of MExos quantity, MExos isolated on ExoBean devices were lysed with RIPA solution and measurements of total protein concentration were obtained by western blot.

### 2.4 Dual Profiling of MCTCs and MExos Using Clinical Samples

#### 2.4.1 Quantitative Analysis in MCTCs and MExos from Clinical Samples

**Figure 4:**
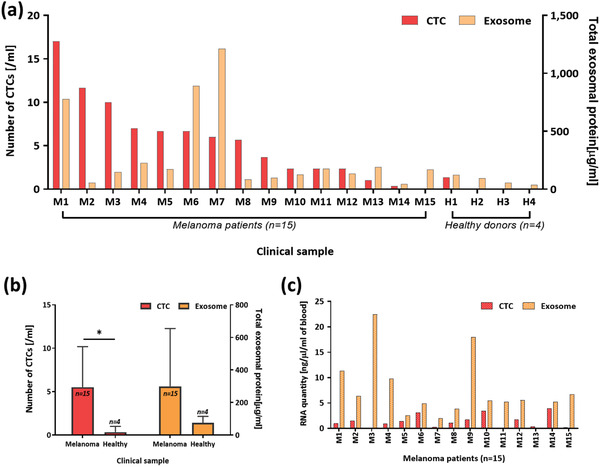
Comparison between results from CTCs and exosomes in melanoma patients; a) comparison of CTC number and total exosomal protein quantity per 1 mL of blood sample; b) average CTC numbers and exosomal protein quantities between melanoma and healthy donors; c) RNA quantities comparison from CTC lysates and exosome lysates.

Quantitative analysis of MCTCs and MExos captured by DUO devices is shown in **Figure** **4**. We first compared the MCTC number and total MExo protein amounts for each sample, normalized to 1 mL of starting blood (Figure 4a). The present devices captured an average of 5.5 MCTCs per mL of blood in melanoma patients, compared to just 0.3 MCTCs per mL in healthy donors (Figure 4b). Total MExo protein from each device is also shown in Figure 4b. Melanoma patients demonstrated significantly more melanoma tumor derived exosomes than healthy donors, averaging 299 µg of protein per mL of plasma compared to only 75.6 µg mL^−1^ in healthy controls. All MCTC number and total exosomal protein quantity results were normalized to 1 mL of blood per sample. Statistical analysis comparing quantity differences in patient samples compared to healthy donors for both MCTCs and MExos was performed using t‐tests. The results of these tests show that the difference in isolated MCTC number was statistically significant (*p*‐value 0.00451), while the difference in protein found using MExos was statistically insignificant (*p*‐value 0.2358). MCTC concentration and total exosomal protein amounts in each patient were analyzed and compared. There is no discernable correlation between MCTC quantity and total exosomal protein amount among those tested. Thus, any test result indicating MCTC concentration or exosomal protein quantity should not serve as a reliable indicator for the other.

#### 2.4.2 Gene Panel Analysis of MCTCs and MExos from Clinical Samples

We next examined RNA quantities from the isolated MCTCs and MExos within each patient. After isolation of either CTCs or exosomes, RLT buffer (Qiagen, USA) was applied to each device for lysis and extraction of RNA. These RNA samples were then analyzed using Bioanalyzer. Results for RNA quantity in captured samples are shown in Figure 4c and are normalized to show RNA per mL of blood for both MCTCs and MExos. For each patient tested, exosome RNA concentration was higher than that found from CTCs. This higher RNA concentration for EVs demonstrates a potential benefit as a marker over CTCs, as less biological fluid is needed for sampling from each patient. As devices with lower fluid requirements yield more practicality in liquid biopsy, exosomes present an exciting alternative to CTCs in this regard. Unpaired *t*‐tests comparing melanoma patient and healthy donor sample sets were then performed for both MCTCs and MExos. As shown in Figure S3 (Supporting Information), these t‐tests returned nonsignificant differences for MCTCs (*p*‐value 0.7413) and MExos (*p*‐value 0.4595). However, these results are likely due to the small sample sizes used for the healthy donors (*n* = 4) compared to clinical samples (*n* = 15) which makes the data more sensitive to error. In future, increasing the number of healthy donor samples may alleviate this sensitivity.

**Figure 5:**
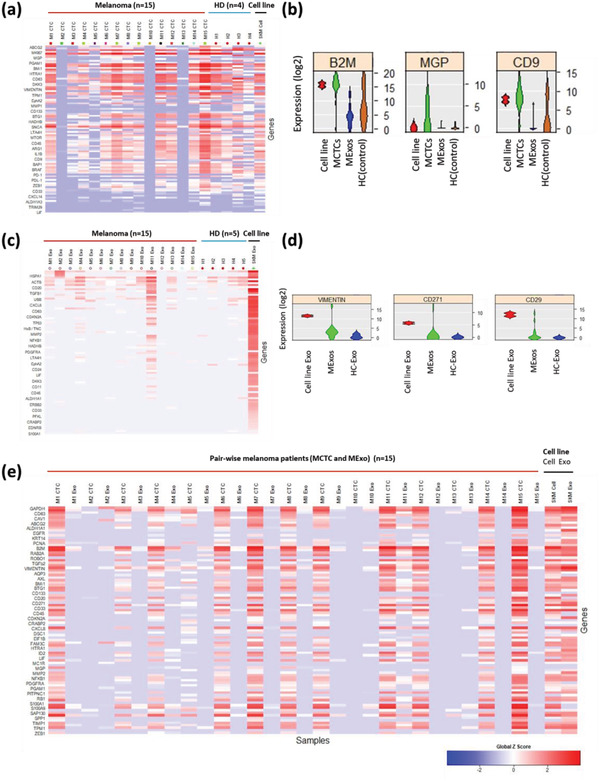
Gene panel analysis of melanoma CTCs (MCTCs) and melanoma exosomes (MExos) isolated by DUO; a) heatmap analysis of gene expression on MCTCs recovered from melanoma patients, healthy donors and melanoma cell line, SK‐MEL‐103; b) violin plot analysis of gene expression on MCTCs; c) heat map analysis of gene expression on MExos recovered from melanoma patients, healthy donors and cell line derived exosomes; d) violin plot analysis of gene expression on Mexos; e) pair‐wise comparison between MCTCs and MExos from same samples.

We then examined specific gene expression levels within each sample. The SINGuLAR platform was used to generate clustered and unclustered heat maps to identify the most significant differentially expressed genes between the CTCs of patient, healthy and control samples (**Figure** **5**a). Violin plots were also generated using the collected expression data and are shown in Figure S7 (Supporting Information). In total, gene expression data were compiled from MCTCs isolated from 15 melanoma patients, 4 healthy donors, and SK‐Mel‐103 cell line as a control. In line with our initial hypothesis and previous research, CTCs highly express standard housekeeping genes, like GAPDH, ACTB and HSPA1. In comparing patient samples with healthy donors (Figure S4, Supporting Information), differences in expression were noted for some genes. In melanoma patient CTCs, *β*2‐microglobulin (B2M) gene expression was nearly treble that found in healthy donor samples. B2M has been linked with the regulation of tumor growth and metastasis in several common cancers.^[^ ^42^ ^]^ Another gene with increased expression in tested patient samples was that of matrix Gla protein (MGP). Increased MGP gene expression has been related to poor patient prognosis in cancers such as breast cancer.^[^ ^43^ ^]^ The plots in Figure 5b show notable MGP expression amongst melanoma patient samples, while no MGP expression was found in any of the healthy donors. We also see a high expression profile for the CD63 gene in our melanoma patients, at nearly double that found in healthy donors. Increased CD63 expression which has been correlated with early stage melanoma tumor progression in other studies. Studies have also shown the expression of CD9 gene to be inversely related to the metastatic potential of melanoma. Our data indicates CD9 expression for the patient samples, however not much can be said about the metastatic potential of the tumor in each case. Some of the other genes on the panel which showed a high expression profile in melanoma patients but not in healthy donors were MTOR, BAP1, CDH1, FAM3C, and TP53.

While significant focus was placed on differences in specific gene expression levels found between melanoma patient and healthy donor CTCs, we also note several genes that showed similar expression levels amongst all CTC samples. For example, a number of examined genes were highly expressed in both patient and healthy donor CTC samples, including CXCL8, S100A8, S100A9, ALDH1A1, and RxRA. This result is noteworthy, as increased expression of each of these five genes has independently been linked to tumor cell growth and/or poor prognosis in various common cancers, including melanoma. In both patient and healthy samples tested, we see a low expression of the gene ALDH1A3, which has been associated with the stemness of both cancer stem cells and normal tissue stem cells. Recent reports reveal that ALDH1A3 is a useful cancer stem cell marker that can be used to enrich tumor‐initiating subpopulations from various cell lines and primary tumors.^[^ ^44^ ^]^ The EGFR gene, which is often associated with lung cancer, had a low expression profile for the CTCs in our study. Low LIF expression levels have been shown to promote cell differentiation, and we observed a very low expression profile for LIF in our CTC patient samples. Some of the other genes which displayed a low expression profile in all samples tested were WNT5A, S100A1, TYRP1, and HxB/TNC.

The gene analysis data for exosome samples was handled in a manner similar to the CTC samples and was tested against the same 96 gene panel with (Figure 5c and Figure S8, Supporting Information). In comparing exosome gene results between patient and healthy donor samples, we find that patient samples had notably more expression of the protein coding gene Vimentin. As shown in Figure 5d, median Vimentin expression in melanoma patient samples was double that of healthy donors. Increased Vimentin expression in epithelial cancers including melanoma has been shown in previous studies, with the protein itself used as a general cancerous exosome marker in cancers such as lung cancer.^[^ ^45^ ^]^ We also noticed a general increase in expression level of CD271 in melanoma patient exosome samples compared to healthy donors. This would make sense as CD271 has previously been proposed as a melanoma marker due to high observed occurrence and indicates increased stemness and tumorigenicity.^[^ ^46^, ^47^ ^]^ Another gene we saw greater expression in patient exosome samples than in healthy donor samples was CD29. This increased expression is noteworthy due to the implication of CD29 in the metastatic diffusion of tumors in previous studies.^[^ ^48^ ^]^ Several genes showed similar expression between healthy donors and patients, such as significant expression of GAPDH, ACTB, B2M, and UBB across most tested samples. We also found no expression of ALDH1A3, EGFR, KRT14, CD11, and PDL‐1 genes in any of the exosome samples from patients or healthy donors. Patient *M11* Exo shows higher gene expressions than the rest of the patients, when compared to the healthy donors’, including genes HSPA1, UBB, and KFB1. HSPA1 is known to be highly expressed in several cancers, including melanoma, and is associated with cancer development and progression. Elevated levels of the UBB gene are shown to be essential for the growth of cancer cells. Here we observed genes S100A9 and S100A8 with high expression profiles. S100A9 has been associated as a key factor in cancer development and tumor spread. While the gene LIF had a low expression in CTCs, we see a very high expression profile for the same gene in exosome samples. FAM3C, GAPDH, Vimentin, and Annexin are a few among the other genes to have high expression profiles. Unlike in the CTC samples, CAV1 and HSPA1 have very low expression in the exosome samples. In comparing results from Figure 5c with total exosome RNA quantity in each sample, there appears to be a correlation between high total RNA quantity and high (gene right above CD20 in the panel) expression.

#### 2.4.3 Correlation between MCTCs and MExos in Melanoma

A comparison between MCTC and MExo gene expression in each patient is displayed in Figure 5e. These results show significantly lower overall gene expression in MExos compared to MCTCs in most patient samples. Exosomes are known for carrying damaged, or degraded mRNA and only contain prepackaged RNA from the cell of origin. However, they are also plentiful and released from all portions of the tumor leading to increased special sampling, whereas the 0–100 CTCs isolated will not represent the entire tumor. Therefore, while the number of genes expressed, and the magnitude differences in log fold change in exosomes may not be as high as CTCs, they offer the potential for a more holistic snapshot of the tumor. There are a couple notable exceptions to this trend, as seen with patient *M11* displaying significant gene expression in both MCTCs and MExos, and in patients *M10* and *M13* who show little to no expression of the entire gene panel within either marker. Another divergence from this trend can be found with the Vimentin gene, which showed similar expression in both CTC and exosome sample across most patients. Similar MCTC and MExo sample gene expression can also be found with B2M and UBB, and ACTB. Overall, gene expression within MCTC patient samples closely resembles that of SK‐MEL‐103 cell line CTCs, while the low gene expression found in patient MExos breaks significantly from the high gene expression displayed on SK‐MEL‐103 cell line derived exosomes. Clinical samples, such as *M8* CTC and *M12* CTC show similar gene expression pattern to exosome cell line SK‐MEL‐103. CD63 and B2M genes are commonly expressed in both the clinical samples and SK‐MEL‐103. B2M gene has functions of cancer cell growth, and CD63 gene has shown to be correlated with cell development and tumor progression.

The present comparison study only used a 96 gene panel that was designed for cellular probing, in fact a different set of genes would likely be found in exosomes compared to cells. Future work would include enhancing our RNA profiling to RNA‐seq, or mRNA microarrays that offer more widespread gene profiling.

## 3 Conclusion

In this study, we have demonstrated the potential of simultaneous isolation of MCTCs and MExos from a single sample using the DUO device. This devices utilization of the MCAM/MCSP antibody cocktail allows for more specific MCTC isolation than offered by widely used single marker methods. The specificity offered by the DUO device allowed us to show that melanoma patients average slightly over 5 MCTCs per mL of blood, while healthy blood contains no MCTCs. Along with capturing MCTCs, the importance of specifically isolating exosomes from melanoma patients is emphasized by our finding that MExos contain a higher RNA concentration than MCTC samples, thus making MExos a potentially more efficient marker. Additionally, using the DUO device, we were able to establish increased MCAM/MCSP expressing exosome protein concentration as a marker for the presence of melanoma. Overall, the ability to isolate MCTCs and MExos with high sensitivity as high throughput from melanoma patient blood samples provides clinicians a powerful and versatile tool for gauging disease progression and treatment response.

## 4 Experimental Section

##### Melanoma Cell Culture and Model Sample Preparation

In order to prepare model samples for melanoma cells, SK‐MEL‐103 cell line was used. The SK‐MEL‐103 cells were cultured in conditioned media and around 5000 cells were spiked into 1 mL of PBS buffer solution or whole blood sample. Besides model samples for MCTCs, cell line derived exosomes were prepared by ultracentrifugation of SK‐MEL‐103 cell culture supernatant with exosome depleted fetal bovine serum. After ultracentrifugation, exosome concentration was measured using NanoSight NS300 (Marven Instruments, UK), and known exosome concentrations were used for model sample preparation.

##### Clinical Sample Preparation

The sample collection and experiments were approved by Ethics committee (Institutional Review Board and Scientific Review Committee) of the University of Michigan. Informed consents were obtained from all participants of this clinical study and melanoma blood samples were obtained after approval by the institutional review board at the University of Michigan (HUM00105509). All experiments were performed in accordance with the approved guidelines and regulations of the ethics committee at the University of Michigan. Whole blood was collected from the University of Michigan cancer center. Roughly 6 mL of whole blood was used for CTC capture per sample, while the remaining blood was processed to collect plasma for exosome isolation. Plasma collection was carried out using 5810R centrifuge (Eppendorf, Germany). Blood was centrifuged at 2000 × *g* for 15 min, allowing for separation of plasma from blood cells. Another centrifugation at 12 000 × *g* was conducted to remove all residual cellular debris. The supernatant plasma sample was filtered through a 200 nm syringe filter and deposited into 2 mL vials. The vials were then stored at −80 °C for future use.

##### DUO Chip Fabrication and Surface Modification for Melanoma

DUO chip fabrication procedure was previously described^[^ ^37^, ^38^ ^]^ and this procedure was followed with modifications for MExo capture. The PDMS mold was bonded to a glass slide using O_2_ plasma treatment (Covance, Femto Science, South Korea). Following bonding, each device was placed on a hot plate at 80 °C for 10 min, then allowed to cool to room temperature. A solution of 500 µL Silane in 5 mL ethanol was injected into each device every 15 min for a total of an hour, followed by a pure ethanol wash. GMBS cross linker solution (14 µL GMBS in 5 mL ethanol) was then injected into the device and incubated for 30 min, followed by another ethanol wash. NeutrAvidin solution (500 µL NeutrAvidin in 5 mL PBS) was injected into each device before the devices were parafilm sealed into Petri dish containers and stored at 4 °C for future use. The prepared DUO devices were conjugated with melanoma associated antibodies MCAM (Miltenyi Biotec, Germany) and MCSP (Novus, USA). 200 µL of antibody solution (2.5 µL MCAM and 2.76 µL MCSP in 250 µL 1%BSA) were injected into each device and incubated 1 h. The devices were then washed with PBS and blocked with either 3% or 1% BSA solution for CTC or exosome capture, respectively. The devices were now prepared to accept samples and capture target CTCs and exosomes.

##### Dual Isolation of CTCs and Exosomes

For CTC isolation, 3 mL of whole blood was slowly applied to each antibody‐conjugated device using an auto‐pump (PHD 2000, Harvard Apparatus, USA) at a flow rate of 5 mL h^−1^. Blood remaining in the devices was then immediately washed out using PBS. Devices were then prepared for either DNA/RNA analysis or immunostaining for imaging. Simultaneously, MExo isolation took place by injecting a 1 mL plasma sample into the device using the syringe pump at a low flow rate of 1 mL h^−1^. Unbound exosomes were then washed out using PBS. Devices with captured exosomes were then prepared for either nucleic acid or protein extraction for sample analysis.

##### Immunostaining of CTCs from Melanoma

After blood samples were applied and washed out, devices were fixed with 1 mL of 4% PFA solution. PFA fixation solution was allowed to incubate for 40 min before being washed out with PBS. Each device was then permeabilized with 1 mL of 0.2% Triton solution and incubated 30 min. Triton was then removed by PBS wash before the application of 1 mL 3% BSA‐2% normal goat serum solution (500 µL 6%BSA, 200 µL normal goat serum, 300 µL PBS), which was incubated for 30 min. Primary staining antibody solution composed of 10 µL anti‐Melan‐A/MART1 (R&D Systems, USA,) 25 µL S100 (mouse IgG2a, ThermoFisher, USA,) and 25 µL CD45 (rat IgG2b, Santa Cruz Biotech, USA) in 1 mL of 1%BSA was pumped into each device and incubated for 1 h. Excess primary antibody solution was removed by PBS wash. A secondary staining antibody solution was then applied containing 5 µL AlexaFluor 546 (goat anti‐mouse IgG2a, Life Technologies, USA) and 5 µL AlexaFluor 488 (goat anti‐rat IgG, Life Technologies, USA) in 1 mL of 1%BSA and allowed to incubate for 1 h in the dark. Excess secondary antibodies were removed by PBS wash followed by the application of 1ml DAPI staining solution (1 µL DAPI in 1 mL 1%BSA.) The DAPI solution was incubated for 15 min followed by a final PBS wash. Devices were imaged using Ti2 microscope (Nikon, Japan) at 10× magnification for cell analysis. Images taken in FITC, DAPI, and CY3 fluorescence.

##### Field Emission Scanning Electron Microscopy (FE‐SEM)

Following exosome isolation, some devices were sampled using a biopsy punch and a razor blade cutter with previously defined SEM sampling procedures,^[^ ^49^ ^]^ with extracted PDMS specimens rinsed with PBS followed by dehydration with increasing concentrations of ethanol (50%, 70%, 90%, 95%, and 100%). The specimens were then incubated with hexamethyldisilazane (Sigma‐Aldrich, USA) in a fume hood overnight to dry. The dehydrated specimens were mounted on SEM stubs with carbon conductive tape and silver paint, and then sputter‐coated with a layer of gold. Exosomes captured by the ExoBean were then examined by FEI Nova 200 Nanolab Dualbeam FIB scanning electron microscope under low beam energies (2.0–5.0 kV) at the Electron Microscopy Analysis Lab (MC2) at University of Michigan.

##### Nanoparticle Tracking Analysis

Evaluation of the exosome concentration and size distribution was analyzed by nanoparticle tracking analysis (NTA) using NanoSight NS300 (Marven Instruments, UK). 30 µL of the initial model sample solution or postcapture sample solution was applied to the jig of the system, and a laser module was mounted inside the main instrument housing. NTA visualizes the scattered lights from the particles of interest based on their Brownian motion. This movement was monitored through a video sequence for 20 s in triplicate. All data acquisition and processing were performed using NanoSight NS300 control software, and concentration of particles in exosome sizes was compared to that of initial samples for calculating capture efficiencies of the ExoBean.

##### Exosomal Total Protein Quantification and Western Blotting

After exosome capture experiments, RIPA buffer (ThermoFisher, USA) was then processed through the device for the lysis of exosomes membrane to harvest the exosomal protein. Total exosomal protein quantity was measured using micro BCA kit (ThermoFisher, USA).

##### Total Nucleic Acid Extraction

The captured cells and vesicles were lysed on chip immediately after PBS washing using RLT buffer (RLT Plus RNeasy Plus lyses, Qiagen, Germany). Inlet and outlet tubing was connected to a sterile 1.5 mL vial and RLT buffer was injected into the inlet. All effluents were stored at −80 °C until RNA analysis. The RNA quantities in the whole lysate was evaluated and 5 ng µL^−1^ of the total RNA for each sample was used to make cDNAs. cDNA synthesis was followed using Cell‐to‐CT kit (Invitrogen, USA) following the manufacturer's protocol. cDNA which was preamplified for the target 96 genes was diluted with 20× GE sample loading reagent (Fluidigm, USA) and used to analyze gene expression by the Biomark HD system (Fluidigm, USA).

##### Melanoma 96‐Gene Panel Expression Analysis for CTCs and Exosomes

The preamplified cDNA was subjected to qPCR to determine expression patterns of target 96 genes, “Melanoma CTC/Exo gene panel,” using TaqMan assays and the Biomark HD instrument. The assay was performed following the manufacturer's protocol with optimizations for this study. After processing, Raw *C*_t_ values generated by Biomark HD (Fluidigm) were analyzed using the SINGuLAR toolset (Fluidigm, USA) and R script to determine the expression pattern of the panel of 96 genes for each sample.^[^ ^50^, ^51^ ^]^ Undetected transcripts automatically generate a *C*_t_ value of 999, which were changed to *C*_t_ of 40 for numerical analyses.^[^ ^50^, ^51^ ^]^ Statistical analysis was performed using R software.

##### SINGuLAR Platform for CTCs and Exosomes

The SINGuLAR Analysis Toolset was chosen to study the gene expression profile of the CTC and exosome patient samples. This platform supports the gene expression analysis on the qRT‐PCR data from the BiomarkHD system. A panel of 96 genes was selected to understand the variations in gene expression between the patient, control and healthy samples. Gene expression data set for each sample is processed according to the guidelines of the SINGuLAR manual before statistical analysis of the data. The raw mRNA expression data from BiomarkHD was grouped and processed in a manner that allows for a thorough comparative study of the single gene expression in CTCs and exosomes for the same patient. The approach adopted in this study also successfully highlights the general trend of gene expression for CTCs and Exosomes and their key variations from the healthy patient samples and the cell line control. Statistical tools of ANOVA and principal component analysis (PCA) were employed to identify the most significant markers out of the 96 genes studied. Heatmap clustering (based on global z score), violin and box plots have been used to visualize this data.

##### Statistical Analysis

All results present as mean ± standard deviation. Statistical analysis was demonstrated using Prism software. Unpaired t‐tests (two‐tailed) were used to compare the differences between total CTCs and exosomal quantities of melanoma patients (*n* = 15) versus healthy controls (*n* = 4). The same statistical test was used for RNA quantity comparison in CTCs and exosomes between melanoma patients (*n* = 15) and healthy controls (*n* = 4). Statistical significance was defined as a two‐tailored *p* < 0.05. Gene expression analysis was conducted using the SINGuLAR Analysis Toolset (Fluidigm), which is operated through *R*. Statistical tools of ANOVA and principal component analysis (PCA) were employed to identify the most significant markers out of the 96 genes studied.

## Conflict of Interest

The authors declare no conflict of interest.

## Supporting information

Supporting Information
