# Supplementary material for: Dual‐Isolation and Profiling of Circulating Tumor Cells and Cancer Exosomes from Blood Samples with Melanoma Using Immunoaffinity‐Based Microfluidic Interfaces
Source: Adv Sci (Weinh). 2020 Aug 19;7(19):2001581. doi: 10.1002/advs.202001581 (PMC7539202; doi:10.1002/advs.202001581)
Supplement: Supplementary file 1 — Supporting Information [file ADVS-7-2001581-s001.pdf]

© 2020 Wiley-VCH GmbH

ADVANCED SCIENCE
Open Access

Supporting Information

for *Adv. Sci.,* DOI: 10.1002/advs.202001581

Dual-Isolation and Profiling of Circulating Tumor Cells and Cancer Exosomes from Blood Samples with Melanoma Using Immunoaffinity-Based Microfluidic Interfaces

Yoon-Tae Kang, Thomas Hadlock, Ting-Wen Lo, Emma Purcell, Anusha Mutukuri, Shamileh Fouladdel, Monica De Silva Raguera, Heather Fairbairn, Vasudha Murlidhar, Alison Durham, Scott A. McLean, and Sunitha Nagrath**

DOI: 10.1002/ ((please add manuscript number))

Supplementary Information

Dual-isolation and profiling of circulating tumor cells and cancer exosomes from blood samples with melanoma using immunoaffinity-based microfluidic interfaces

Yoon-Tae Kang[1], Thomas Hadlock[1], Ting-Wen Lo[1], Emma Purcell[1], Anusha Mutukuri[1], Shamileh Fouladdel[1], Monica De Silva[1], Heather Fairbairn[1], Vasudha Murlidhar[1], Alison Durham[3,4], Scott A. Mclean[2,4,] and Sunitha Nagrath[1,4,*]*

[1]Dr. Yoon-Tae Kang, Thomas Hadlock, Ting-Wen Lo, Emma Purcell, Anusha Mutukuri, Shamileh Fouladdel, Monica De Silva, Heather Fairbairn, Vasudha Murlidhar, Prof. Sunitha Nagrath
Department of Chemical Engineering and Biointerface Institute, University of Michigan, 2800 Plymouth Road, NCRC B10-A184, Ann Arbor, MI 48109, USA
E-mail: snagrath@umich.edu

[2]Dr. Scott A. Mclean
Michigan Medicine Otolaryngology Clinic, 1910 Taubman Center, 1500 E. Medical Center Drive, Ann Arbor, MI 48109, USA

E-mail: scotmcle@med.umich.edu

[3]Dr. Alison Durham

University of Michigan-Michigan Medicine, 1910 Taubman Center, 1500 E. Medical Center Drive, Ann Arbor, MI 48109, USA

[4]Roger Cancer Center, University of Michigan, 1500 E Medical Center Dr. Ann Arbor, 48109

* Corresponding authors

Contents

S1. Melanoma exosome isolation optimization......3

S2. Melanoma exosome isolation optimization......5

S3. 96 Gene panel analysis of melanoma circulating markers 6

S4. Patient information......11

S5. Reagents......12

Figures & Table

Fig. S1. NTA of post capture samples processed by DUO devices 3

Fig. S2. Exosome isolation comparison between various flow rates of sample processing 4

Fig. S3. RNA quantities in CTCs and exosomes from clinical samples 5

Fig. S4. Heat map analysis of mRNA expressions in MCTCs 6

Fig. S5. Heat map analysis of mRNA expressions in MExos 7

Fig. S6. Pair-wise heat map analysis of mRNA expressions in both MCTCs and MExos.8

Fig. S7. Violin plot analysis of mRNA expressions in MCTCs......9

Fig. S8. Violin plot analysis of mRNA expressions in MExos......10

Table. S1. Clinical information of the patient samples 11

Table. S1. Reagents used in this study 12

S1. Melanoma exosome isolation optimization

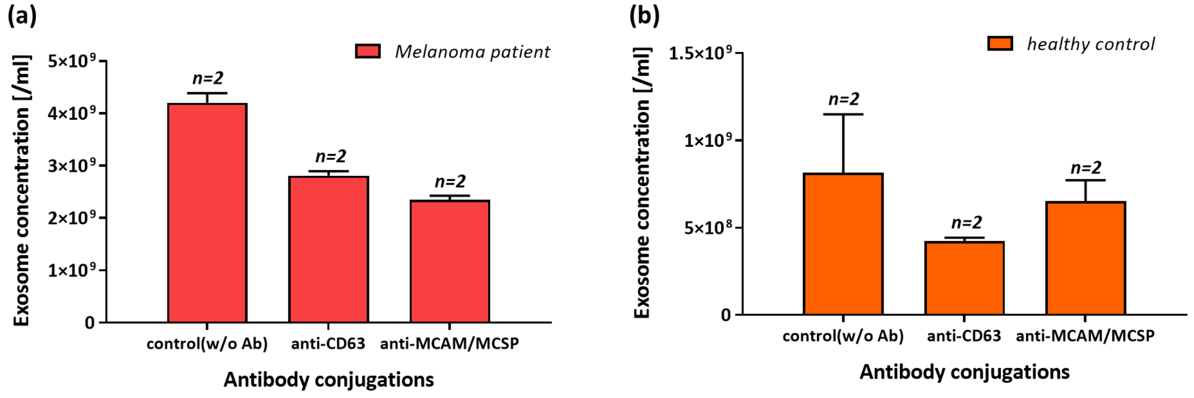

Figure S1. Nanoparticle tracking analysis of post capture samples processed by DUO conjugated without antibodies, with anti-CD63, and with a combination of MCAM and MCSP.

To verify the adequacy of our MCAM/MCSP antibody cocktail in isolating melanoma exosomes, we prepared two different pre-filtered plasma samples from one melanoma patient (M13) and healthy donor. These two samples were processed by three different devices conjugated with antibodies against tetraspanin (anti-CD63), melanoma (anti-MCAM/MCSP), and without antibody (control), and compared. Effluents after a capture event from three devices were analyzed using nanoparticle tracking analysis and concentrations of exosomal size vesicles were compared (**Figure S1**). These results show significant flow though of non-isolated exosomes in control devices, with the best capture performance coming from the device conjugated with anti-MCAM/MCSP for cancer. In healthy donor patients, a lack of melanoma specific proteins on the exosome surface led to antibody cocktail conjugated devices having capture capacities similar to a control device. Devices functionalized with anti-CD63 showed the best capture performance in healthy donor samples, likely due to anti-CD63 being a well-known general exosome marker.

Overall, these results showed that for specific isolation of melanoma exosomes, the MCAM/MCSP antibody cocktail is the optimal choice for our device.

After the optimization of antibodies, we tried finding an optimal flow rate for exosome isolation. Three flow rates, 0.5, 1, and 5ml/h, were tested. All three flow rates demonstrated similar isolation performance, with 1ml/h showing a slightly higher exosome recovery rate in terms of total protein concentration by BCA analysis (**Figure S2**). We therefore chose to use a flow rate of 1ml/hr for the rest of our experiments.

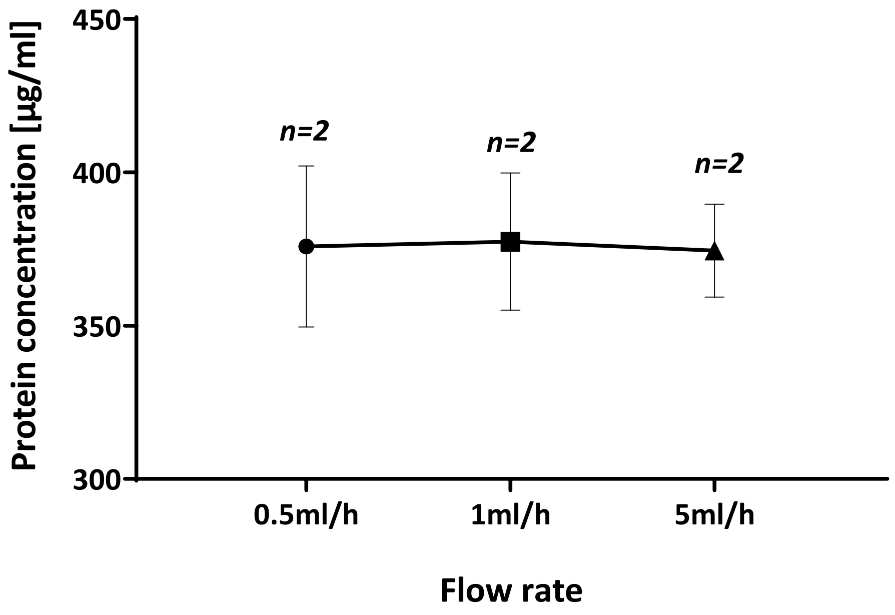

Figure S2. Exosome isolation comparison between various flow rates of sample processing

S2. RNA quantity comparison between melanoma patients and healthy controls

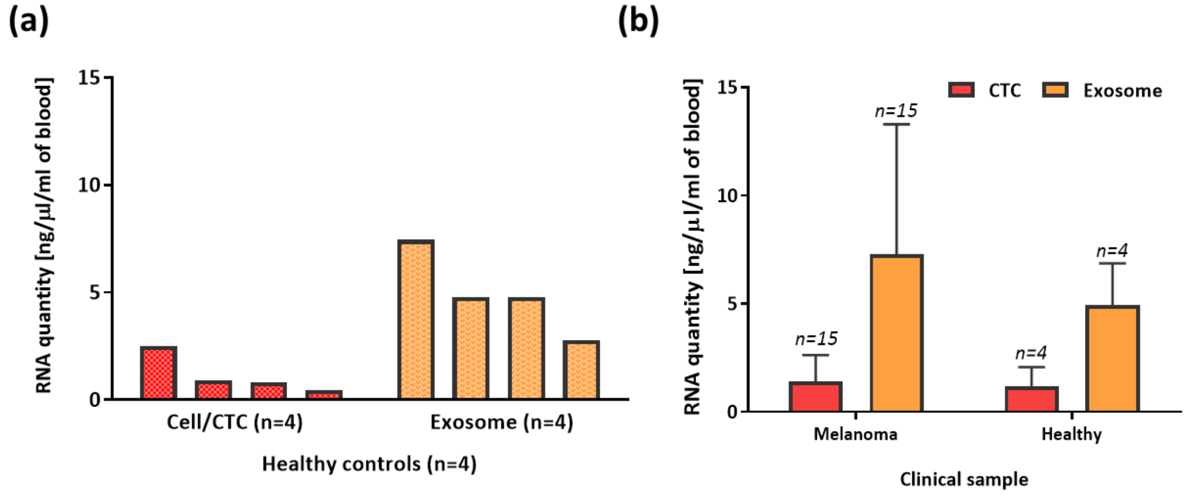

Figure S3. RNA quantities in CTCs and exosomes from clinical samples: a) individual RNA quantities of cells and exosomes in healthy control samples; b) comparison of RNA quantities of CTCs and exosomes in melanoma and healthy control samples

S3. 96 Gene panel analysis of melanoma circulating markers

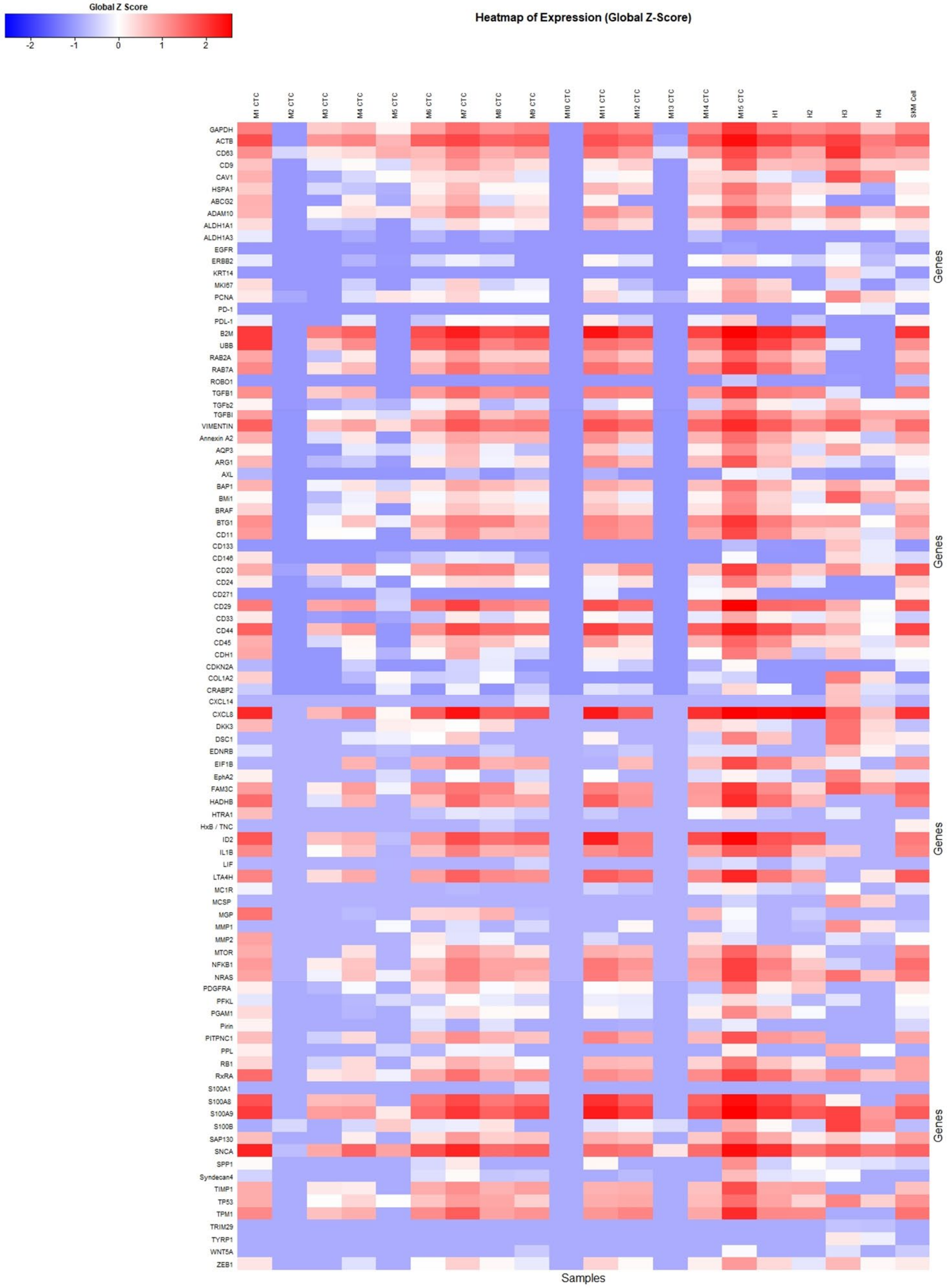

Fig. S4. Heat map analysis of mRNA expressions in MCTCs

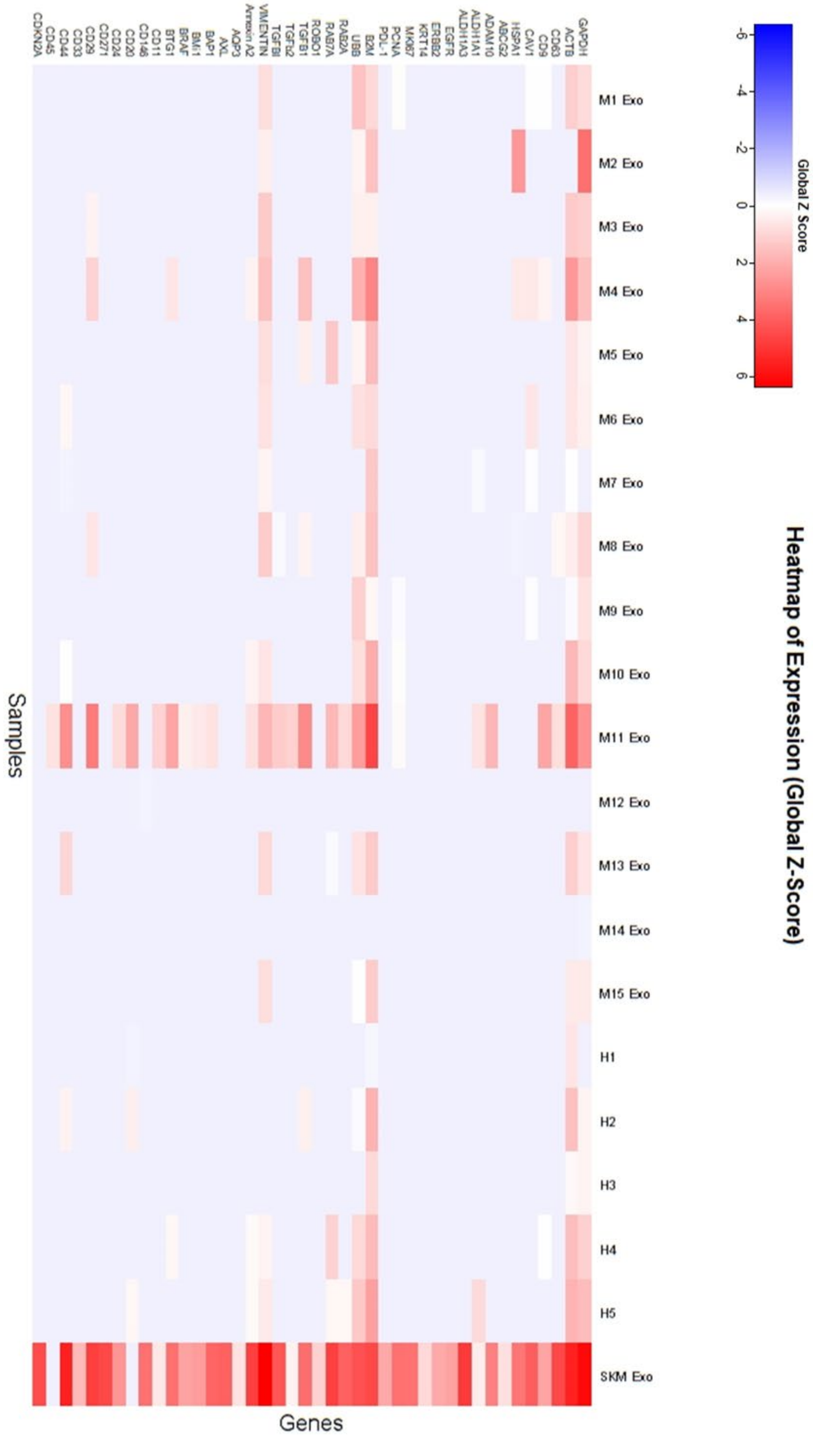

Fig. S5. Heat map analysis of mRNA expressions in MExos

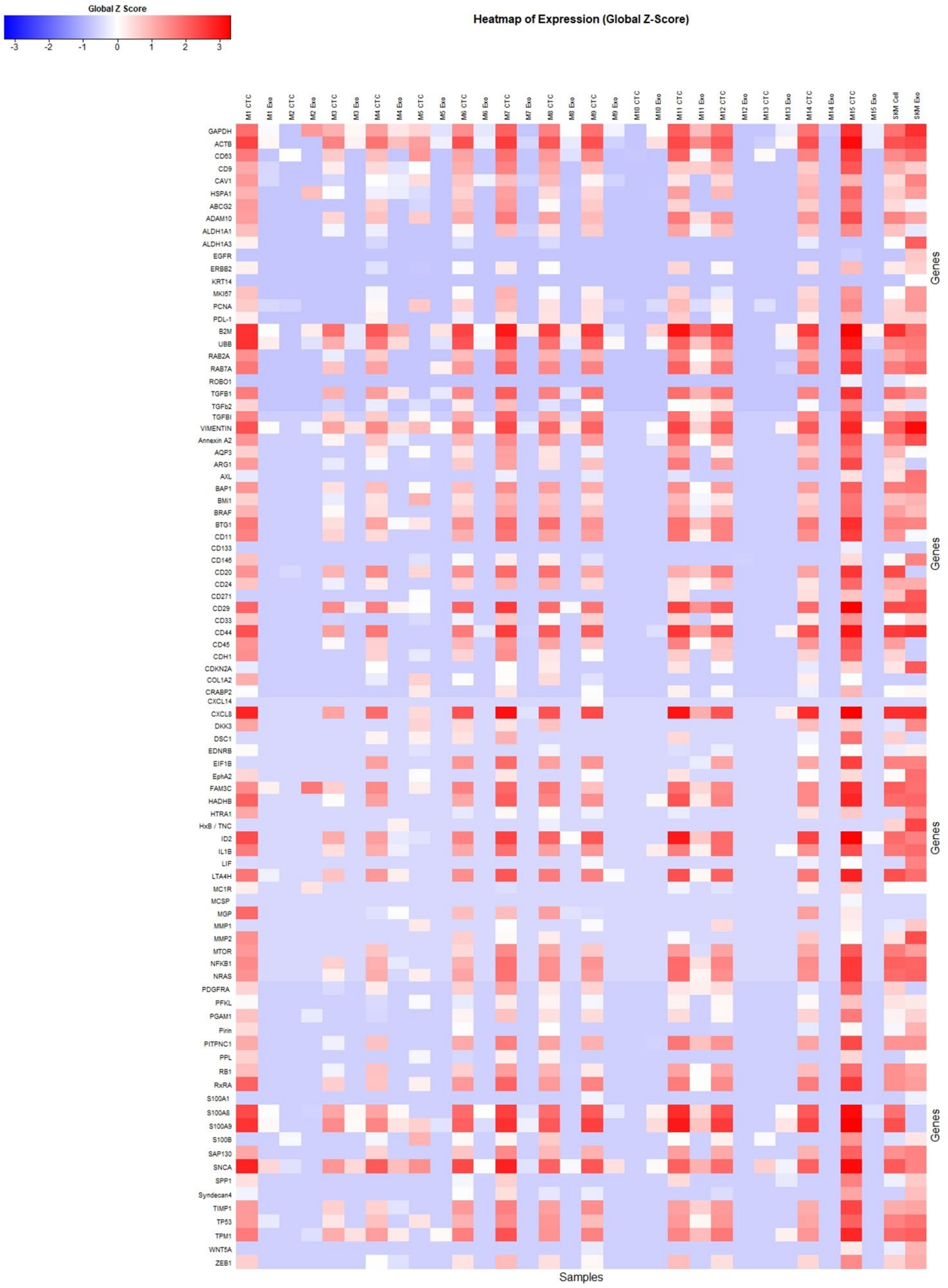

Fig. S6. Pair-wise heat map analysis of mRNA expressions in both MCTCs and MExos

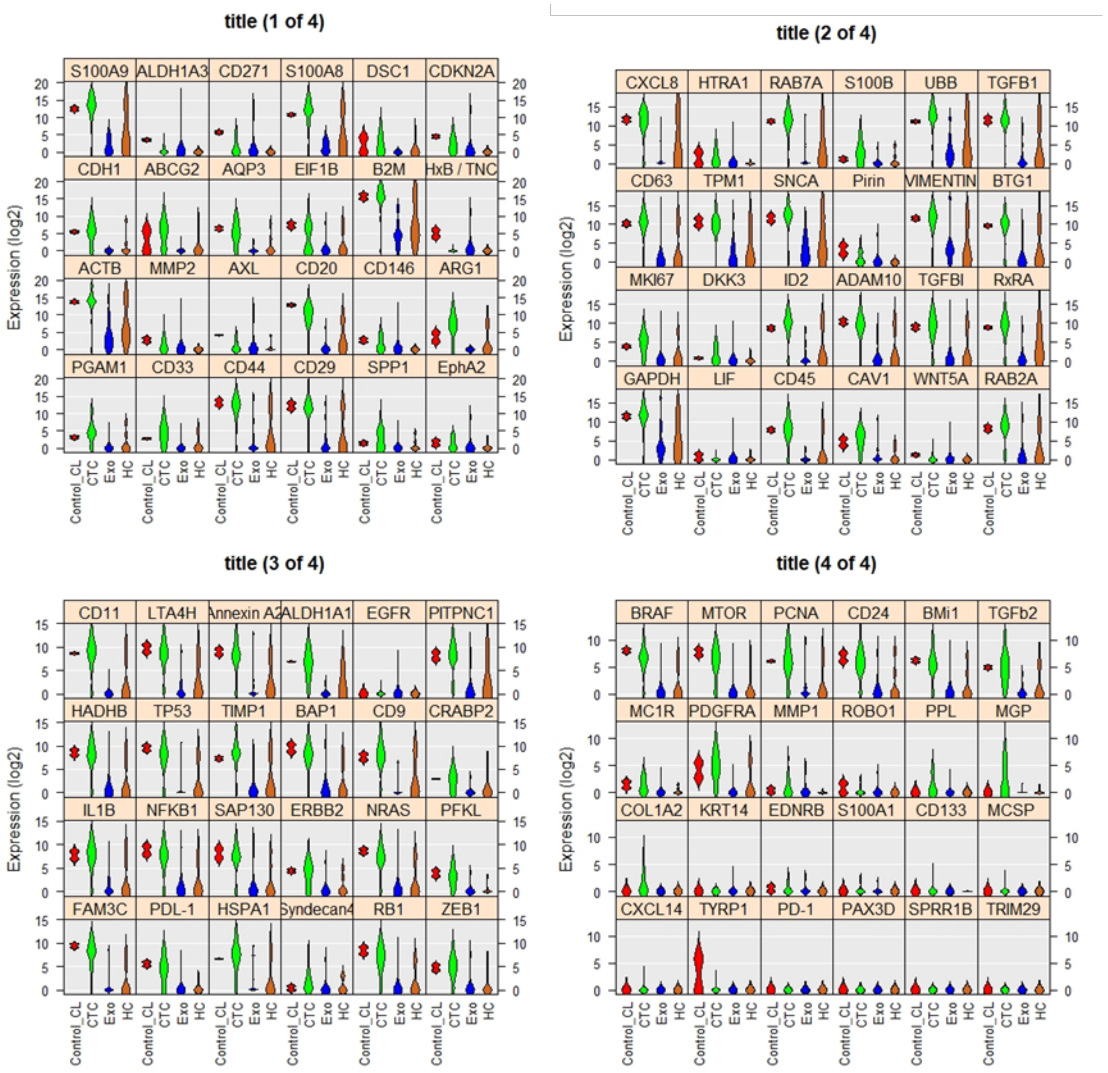

Fig. S7. Violin plot analysis of mRNA expressions in MCTCs

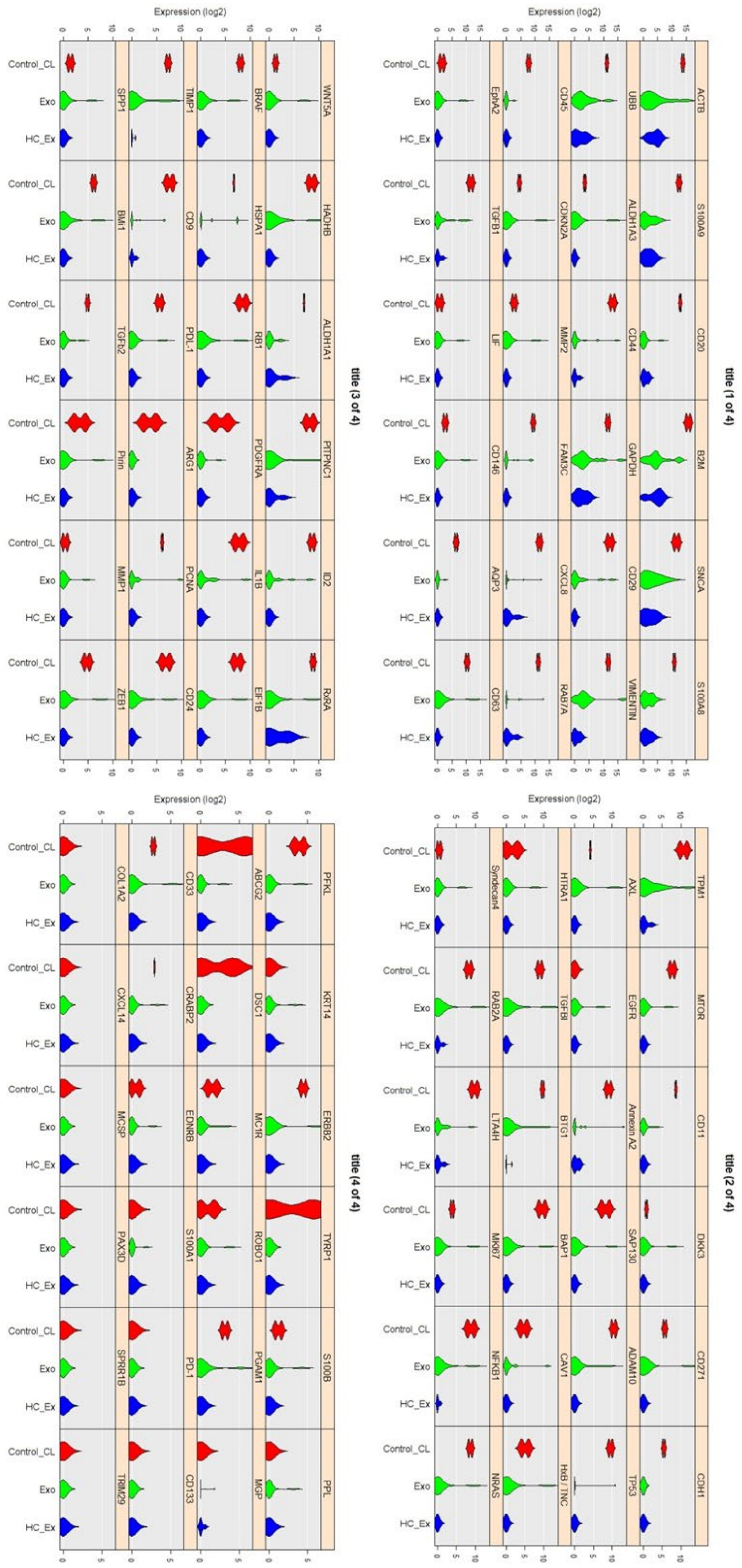

Fig. S8. Violin plot analysis of mRNA expressions in MExos

S4. Patient information

Table S1. Clinical information of the patient samples

Cancer Type	Sample ID	Sample description					
		Sex	Age	Stage	Site	Primary S100	Primary MelanA
Melanoma	***M1***	Male	75	IA	Head & Neck		
	M2	Female	35	IV	Head & Neck		
	M3	Female	77	IB	Head & Neck		
	M4	Female	59	IB	Head & Neck		
	M5	Female	54	IA	Head & Neck		
	M6	Female	77	IB	Head & Neck		
	M7	Male	69	IA	Head & Neck		
	M8	Female	88	IB	Head & Neck		
	M9	Male	80		Head & Neck		
	M10	Male	74	IIIC	Head & Neck	Positive	Positive
	M11	Male	69	IIC	Head & Neck		
	M12	Male	45	IIIC	Head & Neck	Positive	Positive
	M13	Female	80	IIIC	Head & Neck	Positive	Positive
	M14	Male	51	IA	Head & Neck		
	M15	Male	84	IA	Head & Neck		

S5. Reagents

Table S2. The reagents used in this study

Category	Name	Host	Reactivity	Ratio	Catalog number (Company)
**Antibody (capture)**	Biotin-MCAM (CD146)	Mouse	Human	1:100	130-092-852 (MACS)
	Biotin-NG2/MCSP	Goat	Human	1:90	NBP2-45358B (Novus)
**Antibody (IF, primary)**	Melan-A/MART-1	Mouse	Human	1:100	MAB8008 (R&D Systems)
	CD-45	Rat	Human	1:40	SC-70699 (Santa Cruz Bio)
	S100	Mouse	Human	1:40	MA1-26621 (Thermo Fisher)
**Antibody (IF, secondary)**	AF546	Goat	Mouse	1:200	A21133 (Invitrogen)
	AF488	Goat	Rat	1:200	A11006 (Life Tech)
**Dye**	DAPI	-	-	1:1000	
**Buffered solution**	RLT Buffer	-	-		1030963 (Qiagen)
**Etc.**	Pierce RIPA	-	-		89900 (Thermo Scientific)
	Protease Inhibitor Cocktail	-	-		1862209 (Thermo Scientific)
